# Continuous Positive Airway Pressure Device Time to Procurement in a Disadvantaged Population

**DOI:** 10.1155/2015/747906

**Published:** 2015-06-02

**Authors:** Lourdes M. DelRosso, Romy Hoque, Andrew L. Chesson

**Affiliations:** ^1^Department of Neurology, Louisiana State University School of Medicine, Shreveport, LA 71103, USA; ^2^Children's Hospital of Philadelphia, University of Pennsylvania School of Medicine, Philadelphia, PA 19104, USA; ^3^Emory Sleep Center, Department of Neurology, Emory University School of Medicine, Atlanta, GA 30329, USA

## Abstract

*Introduction.* The management of obstructive sleep apnea (OSA) in patients who cannot afford a continuous positive airway pressure (CPAP) device is challenging. In this study we compare time to CPAP procurement in three groups of patients diagnosed with OSA: uninsured subsidized by a humanitarian grant (Group 1), uninsured unsubsidized (Group 2), and those with Medicare or Medicaid (Group 3). We evaluate follow-up and adherence in Group 1. We hypothesize that additional factors, rather than just the ability to obtain CPAP, may uniquely affect follow-up and adherence in uninsured patients.* Methods. *30 patients were in Groups 1 and 2, respectively. 12 patients were in Group 3. Time of CPAP procurement from OSA diagnosis to CPAP initiation was assessed in all groups. CPAP adherence data was collected for Group 1 patients at 1, 3, 6, and 9 months.* Results. *There were no significant differences between groups in gender, age, body mass index, or apnea hypopnea index. The mean time to procurement in Group 1 was shorter compared to Group 2 but not significant. Compared to both Group 1 and Group 2, Group 3 patients had significantly shorter times to device procurement.* Conclusion. *Time to procurement of CPAP was significantly shorter in those with Medicaid/Medicare insurance compared to the uninsured.

## 1. Introduction

Obstructive sleep apnea (OSA) is an increasingly important public health concern associated with an increase in both all cause and cardiovascular related mortalities [1]. Besides its effect on mortality, OSA also has a significant impact on the utilization of medical resources. In years prior to diagnosis and treatment, OSA patients use more physician services and are admitted to hospitals at greater rates compared with individuals without OSA [2]. Fortunately continuous positive airway pressure (CPAP) treats OSA effectively and economically [3].

Cerebrovascular disease and cardiovascular disease are major contributors to health disparity between the insured and the uninsured [4]. Undiagnosed and untreated OSA may be an underrecognized factor, among many other factors, underlying this difference. Treatment with CPAP is the standard of care for patients with OSA. Despite the proven benefit of CPAP use on the cardiovascular consequences of OSA, mortality, and daytime symptoms, CPAP procurement is still a challenge for uninsured populations.

Despite cost-effectiveness and efficacy, CPAP is not available to all patients with OSA. Socioeconomic factors and lack of insurance coverage make it difficult for some to access a CPAP device promptly and effectively. A large retrospective study has shown that OSA patients from healthcare institutions serving the uninsured often fail to follow up after diagnosis, when compared with middle class insured patients [5]. The lack of follow-up in uninsured or underinsured patients may be in part associated with difficulty in obtaining a CPAP device.

In this study we compare time to CPAP procurement and follow-up in three groups of patients with OSA, diagnosed according to the International Classification of Sleep Disorders third edition criteria (ICSD-3) [6], who receive their care at a large medical school teaching hospital whose mission includes caring for the uninsured. The first group consisted of 30 patients without insurance who obtained CPAP through a humanitarian grant. The second group consisted of 30 uninsured patients not covered by the grant, and the third group consisted of 12 patients with Medicaid or Medicare.

## 2. Methods

### 2.1. Groups

Patients in all three groups fulfilled ICSD-3 criteria for OSA and had an overnight in-lab polysomnogram study (PSG) performed at Louisiana State University Health Sciences Center in Shreveport, Louisiana, interpreted by a physician board certified in sleep medicine. All uninsured patients at our institution, who are diagnosed with OSA and who express inability to purchase a CPAP device, are provided with a list of options for CPAP acquisition. The list includes local charities and national charities (American Sleep Apnea Foundation (ASAF) CPAP Assistance Program) and used/donated devices.

Our institution received a humanitarian grant from the American Sleep Medicine Foundation (ASMF) on December 14, 2012. The funds were received on September 5, 2013. The grant was added to our list of CPAP acquisition options on January 2013. The patients had to call, enroll, and schedule an appointment to receive their CPAP devices via the grant.

Group 1 consisted of 30 uninsured patients who received CPAP devices and supplies at no cost through the ASMF grant. The CPAP devices were distributed by a durable medical equipment (DME) company, by the same process the DME company uses with their other patients. Adherence information from device memory cards at 1, 3, 6, and 9 was collected. For those who did not follow up, the factors which might have impeded follow-up were sought and evaluated.

Group 2 consisted of 30 uninsured patients who acquired CPAP via options other than the grant.

Group 3 consisted of 12 patients who had Medicaid or Medicare and were given CPAP prescriptions to be filled out by local DME companies.

### 2.2. Data Acquisition

Demographic data and time to acquisition were collected for all groups. Methods of CPAP acquisition and reasons for lack of CPAP acquisition were also collected for Group 2 and Group 3. Adherence data from CPAP data cards, with adherence defined as percent of nights with device used for more than 4 hours, was collected from Group 1 patients who followed up. Objective quantitative adherence data was not available for patients in Groups 2 and 3.

### 2.3. Statistical Analysis

Nonparametric Analysis of Variance (ANOVA) and Mann-Whitney analysis were used for nonnormal data three-group, and two-group statistics, respectively. Pearson Chi-Square analysis was used for categorical values. Significance was defined as a *p* value < 0.05.

## 3. Results

### 3.1. Demographics

Demographics data of the 3 patient groups is presented in Table 1. Age, body mass index, sex, and race were not significantly different between the groups.

OSA was diagnosed on split-night PSG (the initial diagnostic portion was followed by a positive airway pressure titration), in 26/30 in Group 1, 23/30 in Group 2, and 8/12 in Group 3; the remainder of the patients in each group had separate diagnostic and titration PSGs. Diagnostic apnea hypopnea index (AHI) was not significantly different between the groups, but CPAP pressures were lower for Group 3 (*p* 0.006).

### 3.2. Procurement Data

**Figure 1:**
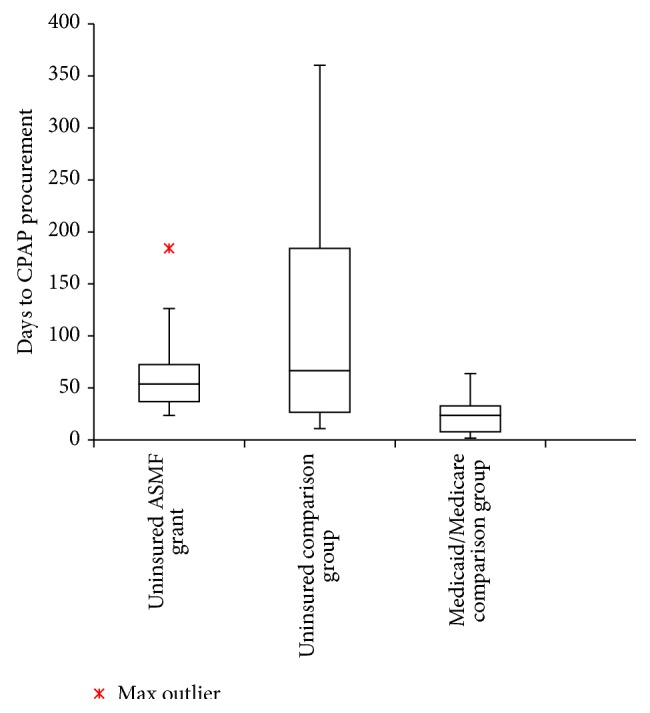
Time to procurement of CPAP devices in three groups: uninsured patients, uninsured patients funded by a charitable grant from the American Sleep Medicine Foundation, and those with government insurance (Medicaid/Medicare). Box and whisker plots of time to procurement of continuous positive airway pressure (CPAP) devices. The lower and upper limits of the boxes delineate the 25th and 75th percentile of the data, the whiskers show the 5th and 95th percentiles, and the horizontal lines show the median. Note the considerable time required for CPAP acquisition in those without insurance coverage, unsubsidized by charitable grants.

Procurement data of the 3 patient groups is presented in Figure 1 and Table 2. All 30 patients in Group 1 received CPAP devices. Factors that affected time to CPAP procurement can be divided into institution dependent factors, time from grant announcement to money distribution and time from diagnostic PSG to patient follow-up, and patient dependent factors, time from patient enrollment to initial CPAP appointment when the device was provided.

**Figure 2:**
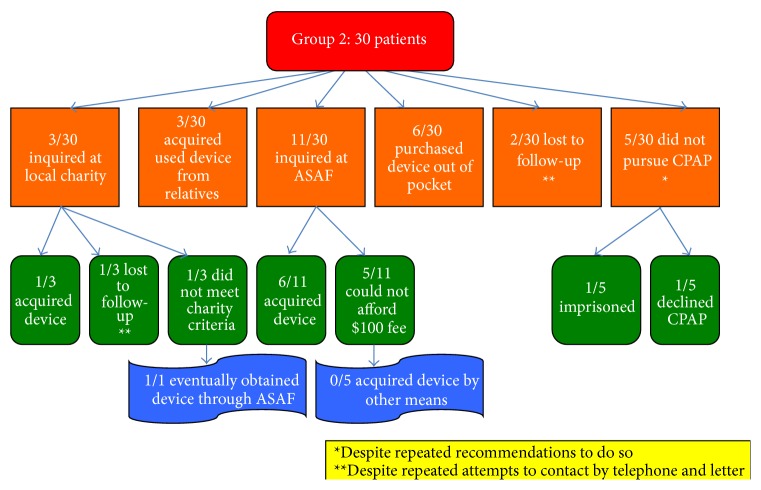
Attempts at CPAP acquisition in 30 disadvantaged, uninsured patients (Group 2). ASAF: American Sleep Apnea Foundation, a charity organization that supplies CPAP devices to those who may not be able to otherwise afford one, for a one-time application fee of $100 at the time of this study.

In Group 2, 16/30 obtained a CPAP device (Figure 2). Patient related factors that affected time to CPAP procurement in Group 2 included: time required to save the money needed for the ASAF fee or purchase of used device, patients who lost telephone connection, patients who did not meet local charity criteria, imprisonment, family support and CPAP refusal.

Of the 12 patients in Group 3, 6 had Medicaid coverage, and 6 had Medicare coverage. 75% of patients in Group 3 acquired a device, compared to 53% in Group 2, with the intergroup difference not reaching significance at a *p* value of 0.19. Of the three Medicaid patients who did not procure a device, one chose not to pursue CPAP therapy; and two did not show up for follow-up appointments, could not be contacted by telephone, and did not respond to letters mailed to their home address. There were no other factors identified affecting CPAP procurement time in Group 3.

Though status of CPAP acquisition was not significantly different between Group 2 and Group 3, time to procurement was significantly different between these two groups with a *p* value of 0.02.

The mean time to procurement in Group 1 was shorter compared to Group 2 but not statistically significant with a *p* value of 0.72. Compared to both Group 1 and Group 2, Group 3 patients had significantly shorter times to device procurement with a *p* value of 0.006.

### 3.3. Follow-Up Data Group 1

All patients in Group 1 were given follow-up appointments at 1, 3, 6, and 9 months for adherence evaluation. 1/30 died prior to follow-up from complications not associated with OSA, 1/30 was imprisoned after being supplied with a CPAP, and 8/30 other patients did not follow up after acquisition of CPAP.

At 3 months 12/30 did not respond to telephone calls; 8/30 did not have working telephone numbers, did not respond to letters, and their address could not be verified. At 9 months 11/30 did not respond to telephone calls; 6/30 had disconnected telephone calls and did not respond to letters; and 1/30 moved out of state without leaving a forwarding address. Of the 20/30 who followed up at least once, 11/30 followed up only once, 4/30 followed up twice, 1/30 followed up for three visits, and 4/30 followed up for all four scheduled visits. Significant factors identified to impede follow-up included telephone disconnection and lack of transportation.

### 3.4. CPAP Adherence Data Group 1

Adherence was 40.4% (±22.8) for the 9/30 who followed up at 1 month, 34.2% (±28.3) for the 10/30 who followed up at 3 months, 33.5% (±18.3) for the 7/30 who followed up at 6 months, and 24.9% (±22.5) for the 11 who followed up at 9 months.

Adherence at 1-month follow-up was 28.8% (±15.3) in 6 black patients, compared to 63.2 (±18.2) in 3 white patients. Adherence at 9-month follow-up was 28.2 (±32.7) in 4 black patients, compared to 23.1 (±17.3) in 7 white patients. One-month adherence differed significantly between blacks and whites with a *p* value of 0.02 but did not differ significantly at 9 months with a *p* value of 0.85.

There were no significant differences in adherence based on AHI at 1 month, 3 months, 6 months, or 9 months.

## 4. Discussion

According to the Centers for Disease Control Health Disparities and Inequalities Report from 2013, avoidable health inequalities in the United States are in part due to gaps in access to healthcare and treatment for socioeconomically disadvantaged populations [7]. For example, according to the Centers for Disease Control between 2007 and 2010 the prevalence of obesity, an important driver for OSA, significantly increased in the United States, with substantial disparities based on socioeconomic status.

Our study exemplifies some of the difficulties in seeking healthcare faced by the socioeconomically disadvantaged, through the process of providing CPAP devices and CPAP follow-up to a group of uninsured and underinsured patients, even when CPAP devices are provided at no cost. Prior studies have shown that only half of patients use CPAP for more than 4 hours a night, and CPAP adherence in those with low socioeconomic status is 34% while adherence in those with high socioeconomic status is 62% [8]. Our study on uninsured patients yielded comparable results, with adherence of 40.4% at 1 month and 24.9% at 9 months. Studies have shown that black race and low socioeconomic status are predictors of low adherence [9]. In our study, though whites had higher rates of CPAP adherence at 1-month follow-up, adherence rates at 9-month follow-up were similar between uninsured black and white patients.

Limitations of our study include the limited number of patients in all the three groups and the assessment of patients all being at a single institution. Strengths of our study compared to prior work are that board certified sleep medicine physicians assessed all patients and all patients received in-lab full night PSG and other social problems were explored.

Follow-up was a challenge in the uninsured, with some of the largest hurdles being disconnected telephone numbers and unanswered telephone calls. Other social problems contribute to lack of compliance (imprisonment, lack of utilities or housing stability, and economic problems with returning to clinic for education and problem solving for difficulties).

In conclusion, time to CPAP procurement was shorter in patients with Medicaid or Medicare insurance when compared to uninsured patients that acquire CPAP from charity organizations or purchase devices by themselves. CPAP acquisition via sources other than insurance is influenced by institution specific factors (requirements, cost, and time to disbursement) and patient specific factors (telephone, ability to fill out forms, and lack of transportation).

We also found out that providing CPAP devices to uninsured populations through a humanitarian grant does not by itself solve the adherence and follow-up challenges seen in the general population and this population may have additional adherence barriers that need to be further explored. For those with limited resources, acquisition of and adherence with CPAP therapy often require profound motivation and determination and are fraught with difficulty.

## Figures and Tables

**Table 1:** Demographics.

Variable	ASMF grant Group 1	Uninsured Group 2	Medicaid/Medicare Group 3	Three-group *p* value
Total	30	30	12	NA
Age: mean ± SD	47 ± 9	46 ± 10	52 ± 13	0.32
BMI: mean ± SD	39 ± 9	39 ± 9	38 ± 5	0.81
Men: *n* (%)	10 (33)	14 (47)	6 (50)	0.30
Black: *n* (%)	18 (60)	13 (43)	4 (33)	0.39
White: *n* (%)	11 (37)	16 (53)	8 (67)	NA
Hispanic: *n* (%)	0	1 (3)	0	NA
Asian: *n* (%)	1 (3)	0	0	NA
AHI: mean ± SD	32 ± 35	46 ± 44	32 ± 27	0.34
CPAP pressure: mean ± SD	13 ± 4	12 ± 4	9 ± 2	0.006
CPAP procurement: *n* (%)	30 (100)	16 (53)	9 (75)	0.0001

BMI: body mass index, AHI: apnea hypopnea index, and CPAP: continuous positive airway pressure.

**Table 2:** Procurement data.

CPAP procurement method	Number (%)	Days to procurement: mean ± SD
Group 1	30 (100)	62 ± 35
Group 2		
(i) Total	16 (53)	113 ± 113
(ii) ASAF	6 (20)	135 ± 123
(iii) Local charity	1 (3)	11
(iv) Purchased by patient	6 (20)	144 ± 128
(v) Purchased by relative	2 (7)	56 ± 47
(vi) Device donated by family member	1 (3)	15
Group 3	9 (75)	25 ± 9

ASMF: American Sleep Medicine Foundation, CPAP: continuous positive airway pressure (CPAP).
